# Pulseometry: A differential readout to compensate signal drift of potentiometric probes

**DOI:** 10.1126/sciadv.aef5993

**Published:** 2026-08-21

**Authors:** Yaotian Wu, Junyu Zhou, Yu Qin, Eric Bakker

**Affiliations:** ^1^ Department of Inorganic and Analytical Chemistry, University of Geneva, Quai Ernest-Ansermet 30, CH-1211 Geneva, Switzerland.; ^2^ Eaglenos Sciences Inc., B2-2, Treehouse Headquarters Cluster, No. 73 Tanmi Road, Nanjing Jiangbei New Area, Nanjing, Jiangsu 210044, China.

## Abstract

Ion selective electrodes are widely used sensors in point-of-care testing devices to assay electrolytes in whole blood samples. With single-use system, the first solution contact normally results in an important signal drift that negatively affects measurement accuracy. In this work, we propose an approach coined pulseometry to compensate for signal drift. The potentiometric signal is processed through a differential circuit incorporating RC components of different time constants. The differential potential will remain constant for a slowly drifting baseline, while a rapid potential change will result in a peak-shaped difference signal that may be baseline subtracted and integrated to extract the components information. The method was successfully integrated into mass fabricated, microfluidic test cards used in commercial blood gas electrolyte analyzers. Blood calcium levels from three individuals were measured, giving SDs of 0.008 to 0.024 mM, improving precision by 36 to 67% compared to potentiometry. These results showcase the potential of pulseometry for improving the performance of electrochemical sensing in clinical diagnostics.

## INTRODUCTION

Point-of-care testing (POCT) refers to rapid clinical testing that can be performed at the patient’s bedside (*1*). POCT typically features a user-friendly protocol with minimal sample processing and consumption, with the personnel conducting the test requiring minimal training. Over the past decades, POCT devices integrating microfluidics and techniques such as flow cytometry (*2*, *3*), immunoassays (*4*–*6*), enzymatic biosensors (*7*, *8*), and ion-selective sensors (*9*–*12*) have been developed and implemented into these devices. This allows one to detect biomarkers (*13*, *14*) and physiological parameters (pH, Na, K, Ca, glucose, etc.) (*15*–*17*) successfully in undiluted whole blood. The blood gas and electrolyte analyzer is a representative example of modern POCT devices in which the blood sample is assayed by integrated electrochemical sensors (*18*). Among the target parameters, blood chloride (*19*), calcium (*20*), magnesium (*21*), potassium (*22*), sodium (*23*), pH (*23*), and carbon dioxide (*24*) are quantified potentiometrically by an on-chip array of ion-selective electrodes (ISEs).

The potentiometric response of an ISE originates from changes in the phase boundary potential at the interface between the ISE membrane and the contacting solution (*25*). The membrane is composed of a matrix material doped with an ionophore, which provides ion selectivity, and an ion exchanger, which consists of lipophilic ions of opposite charge to stabilize the target ion in the membrane (*26*). Ideally, the phase boundary potential may be described by the Nernst equation. For a calcium-selective electrode (Ca-ISE) it is given by

$$\Delta \phi_{\text{membrane}}^{\text{aq}}=\frac{\mu_{Ca^{2+}}^{0,\text{aq}}-\mu_{Ca^{2+}}^{0,\text{org}}}{2F}+\frac{\mathrm{RT}}{2F}\text{ln}\frac{a_{Ca^{2+}}^{\text{aq}}}{a_{Ca^{2+}}^{\text{org}}}$$ (1)

where $\mu_{Ca^{2+}}^{0,\text{aq}}$ and $\mu_{Ca^{2+}}^{0,\text{org}}$ is the standard electrochemical potential of the Ca^2+^ in the aqueous and membrane phase, F is the Faraday constant, R is the gas constant, T is the absolute temperature, $a_{Ca^{2+}}^{\text{aq}}$ and $a_{Ca^{2+}}^{\text{org}}$ are the activities of Ca^2+^ in the aqueous and organic membrane phase, and 2 is the valency of Ca^2+^.

The ISE may give a stable potential response if the counter ion of the membrane ion-exchanger is the same as the analyte ion and independent of sample composition. Unfortunately, ion exchangers are not commercially available with every desired counter ion such as Ca^2+^, which would be required for a calcium probe. To replace the original counter ion with Ca^2+^, a conditioning step is normally performed to stabilize the membrane potential. For this purpose, the ISE may be immersed in a solution containing Ca^2+^ salt for a prolonged period of time, during which exhaustive exchange with the original counter ion is allowed to take place (*26*, *27*). Unfortunately, such a step is not practical for mass fabricated single use sensors. Additional sources of signal drift include the hydration of the membrane and the formation of a water layer inside the solid contact ISE (*28*, *29*). Since single-use microfluidic cartridges of blood gas analyzer are designed to work out of the box, signal drift will invariably be observed during measurement and may negatively affect precision and accuracy.

**Fig. 1.**
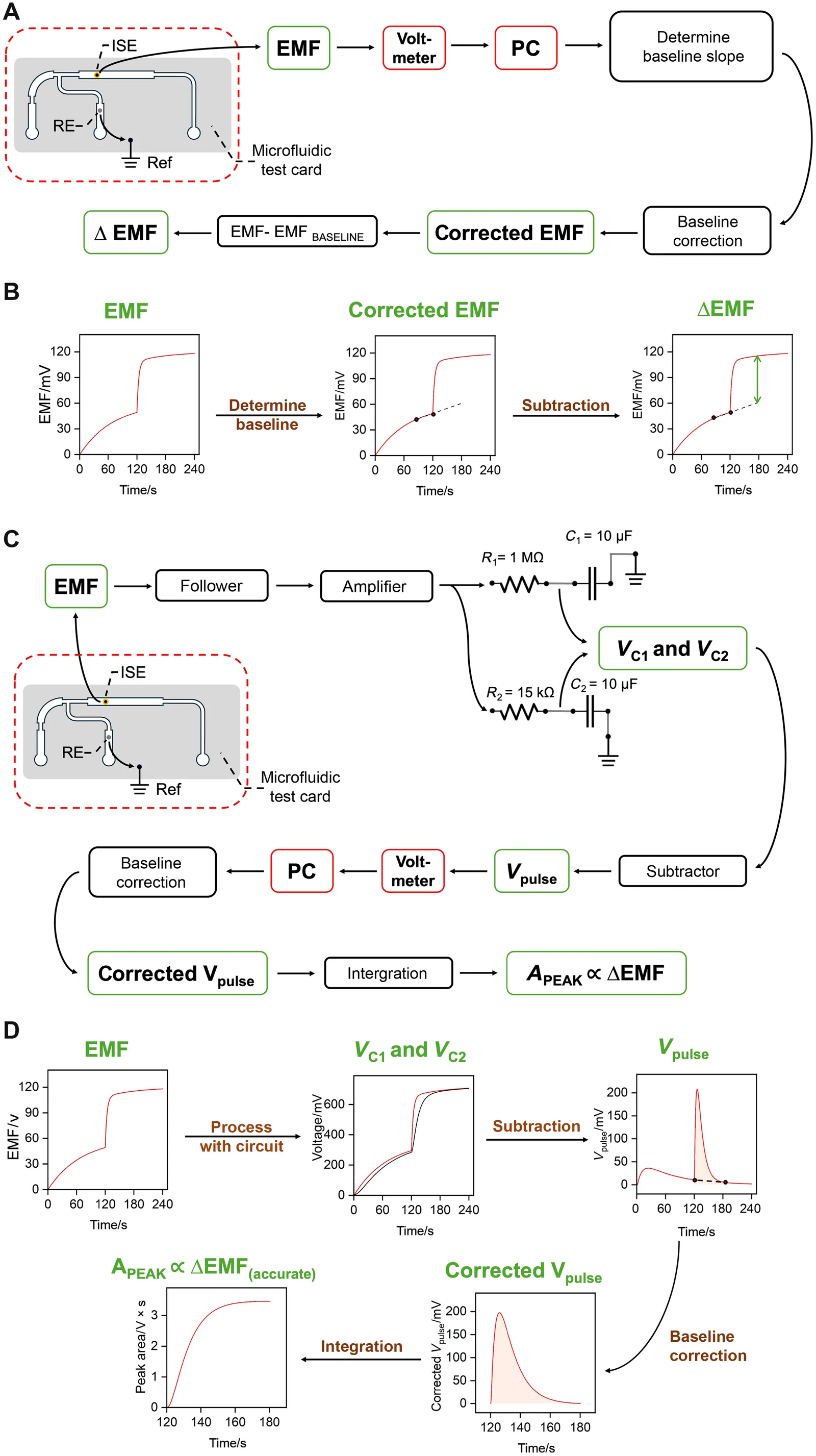
Principle of the new approach. (**A**) Block diagram of the data treatment of traditional blood gas electrolyte analyzers. In this example, the sample is injected and displaces the reference solution at 120 s. If the signal drifts changes after injection of sample as shown, then the resulting error may be considerable. (**B**) Schematic of observed signal change in potentiometry upon sample injection. The initial drift is extended to form the baseline for the measurement of the sample, which results in an error in this example. (**C**) Block diagram of the proposed pulseometry approach. The amplitude of the $V_{\text{pulse}}$ baseline corresponds to the drift rates of the EMF signal. The error attributed to the variation of drift may be partially compensated by baseline correction to obtain a more accurate observed ΔEMF value. (**D**) Data treatment demonstration of the pulseometry approach: The signal is loaded onto two RC elements of different time constants and subtracted to give a peak-shaped signal that can be baseline corrected and integrated with time. Peak area scales linearly with potential change (see the “Characterization of the circuit” section).

For the reasons given above, blood gas analyzers require calibration and appropriate data treatment of the electromotive force (EMF) signal from unconditioned ISEs that exhibit drift (*30*). Sample concentrations are assessed by measuring the potential difference for a standard solution of known composition and the blood sample injected sequentially. The drifting potential signal of the sensor in contact with standard, before sample injection, may be virtually extended in time to serve as baseline for the sample measurement and improve precision (Fig. 1, A and B). The error originating from signal drift may however only be adequately compensated if the rate remains constant. An important change in potential drift may amplify the error.

To achieve improved precision, constant potential coulometry was introduced by Bobacka’s group in 2016 as a possible alternative to zero current potentiometry and further developed (*12*, *31*–*37*). Here, the cell potential is imposed externally, and the potential change at the ISE results in the charging of a capacitive element. While excellent precision has been reported (*35*), the method requires stable potential signals to work properly. It is not designed to compensate for drift.

In this study, an approach is introduced to process the potentiometric signal drifts at varying rates. A voltage follower is first used to avoid passing current through the measurement cell. The EMF is then amplified and applied to charge two parallel RC pairs of different time constants (see Fig. 1, C and D). Upon excursion of the voltage, one capacitor will be charged faster than the other, and we may observe the difference with a subtractor. The faster the applied voltage varies, the larger this difference will be. Thus, the output of this subtractor may indicate the slope of the EMF signal. Upon injection of the sample solution, we may assume that the potential signal changes much faster than the underlying drift. In this manner, a peak-shaped subtracted signal may be obtained upon injection of sample solution. After subtracting the baseline, the potential drift may be practically eliminated and result in improved precision.

## RESULTS

### Pulseometry circuit

The EMF provided by the ISE against a reference electrode (RE) may be applied to charge capacitive devices (*38*, *39*). However, charging a capacitor directly with an ISE would result in charge transport through the electrode and in undesired polarization of the indicator electrode (*34*, *36*, *37*). It is well established that isolating the ISE circuit from the other electronic components with a voltage follower avoids polarization, which was adopted here (*35*). The electromotive force of the ISE measurement cell was first buffered by a voltage follower. To reduce high frequency noise, an RC low pass filter with RC = 0.1 s was used to condition the output signal from the voltage follower. To improve the signal-to-noise ratio, the EMF signal was then amplified 5.7 times before charging the two RC pairs. The capacitor voltage for the RC element with the short time constant τ_R1C1_ = 0.15 s is designed to follow the ISE signal in real time. For the second RC pair, however, a larger time constant of τ_R2C2_ = 10 s was chosen to give a delay for a rapidly changing EMF signal. With a constant EMF, no voltage difference may be observed between C1 and C2. For an EMF drifting at a constant rate, the voltage difference will instead assume a nonzero but constant value. A rapid change of EMF upon injection of sample should now result in a transient voltage difference, since C1 and C2 will charge at different rates. This difference should decay back to zero when the two capacitors are eventually charged to the same voltage. In the instrument, the voltage difference between the two capacitive elements was measured by an instrumentation amplifier (subtractor). Of course, the resistance, capacitance, and gain of the amplifier may be tuned to optimize the signal according to the specific application. The scheme of the circuit is illustrated in fig. S1.

### Characterization of the circuit

The pulseometry approach was developed to detect sudden EMF changes and distinguish them from continuous voltage drifts. The associated circuit was initially simulated using a step signal that simulates an immediate EMF change. The voltage change across a capacitor in an RC circuit during the charging process can be described by

$$V_{C}\left(t\right)=V_{0}+G\Delta V\left(1-e^{-\frac{t}{\text{RC}}}\right)$$ (2)

where $V_{C}\left(t\right)$ is the voltage over the capacitor, $V_{0}$ is the initial voltage of the capacitor before the charging event, ΔV is the amplitude of the imposed voltage change, *G* is the total gain of the signal amplification, *t* is the charging time, *R* is the resistance, and *C* is the capacitance.

When measuring the voltage difference between C1 and C2, the output of the instrumental amplifier $V_{\text{pulse}}\left(t\right)$ can then be expressed as

$$V_{\text{pulse}}\left(t\right)=V_{C_{1}}\left(t\right)-V_{C_{2}}\left(t\right)=G\Delta V\left(1-e^{-\frac{t}{R_{2}C_{2}}}\right)-G\Delta V\left(1-e^{-\frac{t}{R_{1}C_{1}}}\right)$$ (3)

The integrated voltage over time in the interval from 0 to *T* is given express as *A*

$$A=\int_{0}^{T}V_{\text{pulse}}\left(t\right)\mathrm{dt}=G\Delta V\int_{0}^{T}\left(e^{-\frac{t}{R_{1}C_{1}}}-e^{-\frac{t}{R_{2}C_{2}}}\right)\;\mathrm{dt}$$ (4)

Assuming that the integration time *T* exceeds 5 τ, the integral $\int_{0}^{T}\left(e^{-\frac{t}{R_{1}C_{1}}}-e^{-\frac{t}{R_{2}C_{2}}}\right)$ can be understood as a constant $k=G\int_{0}^{T}$$\left(e^{-\frac{t}{R_{1}C_{1}}}-e^{-\frac{t}{R_{2}C_{2}}}\right)$. This leads to the simplified relationship

A=kΔV (5)

**Fig. 2.**
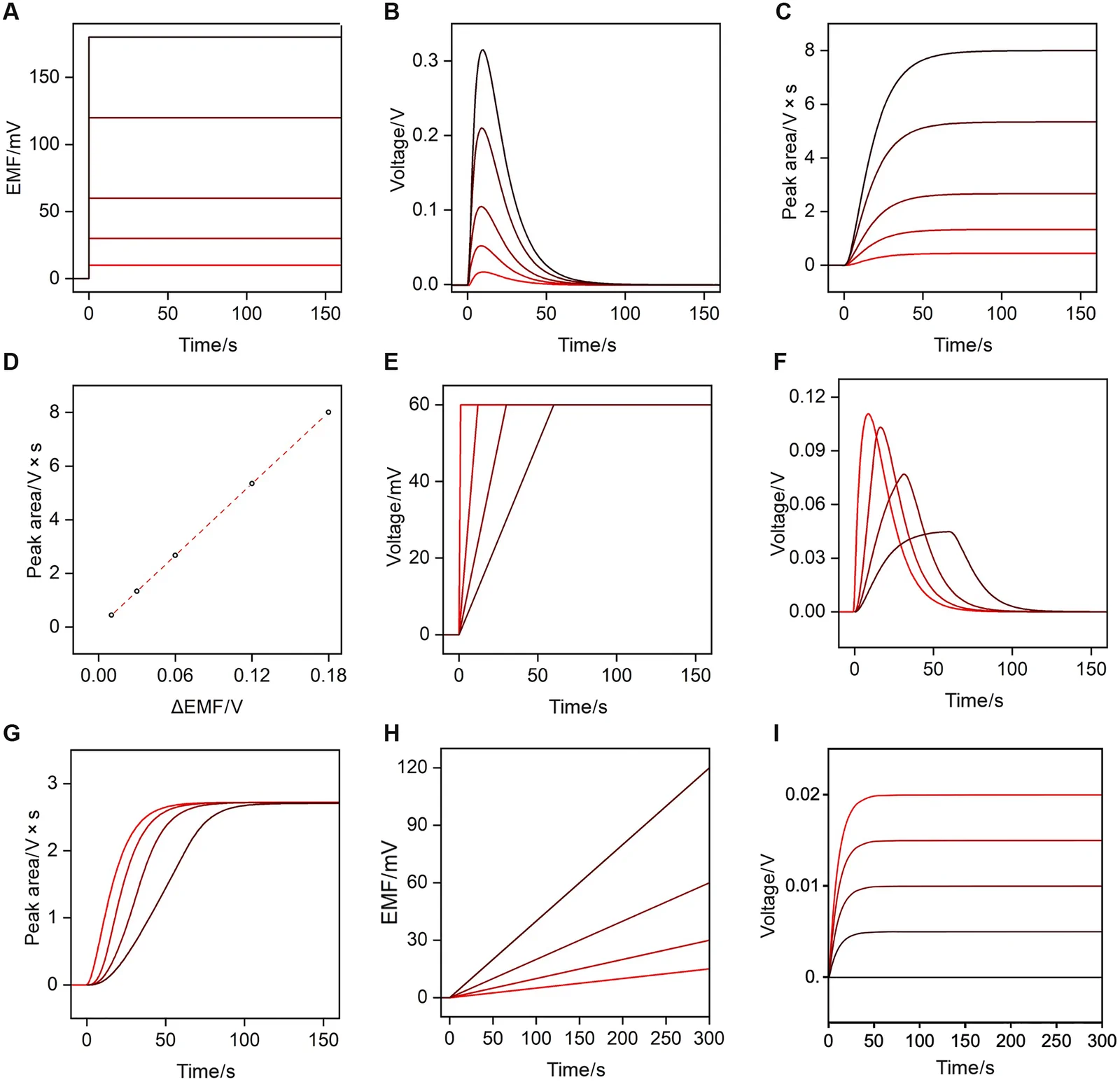
Characterization of the circuit. (**A**) Potential step changes generated by a Keithley 2450 source meter of different amplitudes generate (**B**) pulse-like signals with (**C**) different resulting peak areas. (**D**) Peak area is found to be proportional to the amplitude of the voltage change. Red line: numerical simulation by software; black line: experimental data. (**E**) Imposing voltage signals that change at different rates give (**F**) a pulse-like signal as the output, for which (**G**) the integrated peak areas of the output signal are all the same. (**H**) Input voltage signals that continuously drift at different rates give (**I**) a baseline shift of the integrated difference signal.

The proportionality between the integrated voltage difference over time and imposed EMF was validated experimentally as shown in Fig. 2 (A to D). This relationship facilitates conversion between pulseometry readings and potentiometric measurements. With sufficiently long times, the constant depends only on the amplifier gain and the chosen time constants. A source measurement unit instrument provided imposed experimental voltage changes, allowing for the measurement of $V_{\text{pulse}}$. To emulate the EMF variations occurring during sample injection, different rates of linear voltage changes were applied to the circuit (see Fig. 2E), giving the transient voltage differences shown in Fig. 2F. It was observed that the resulting integrated pulse area remained constant (Fig. 2G), independent of the rate of EMF change. The impact of continuous EMF drift (Fig. 2H) was investigated in Fig. 2I. The pulseometry output was found to stabilize after ∼5 τ, allowing for baseline correction to give improved measurement accuracy.

**Fig. 3.**
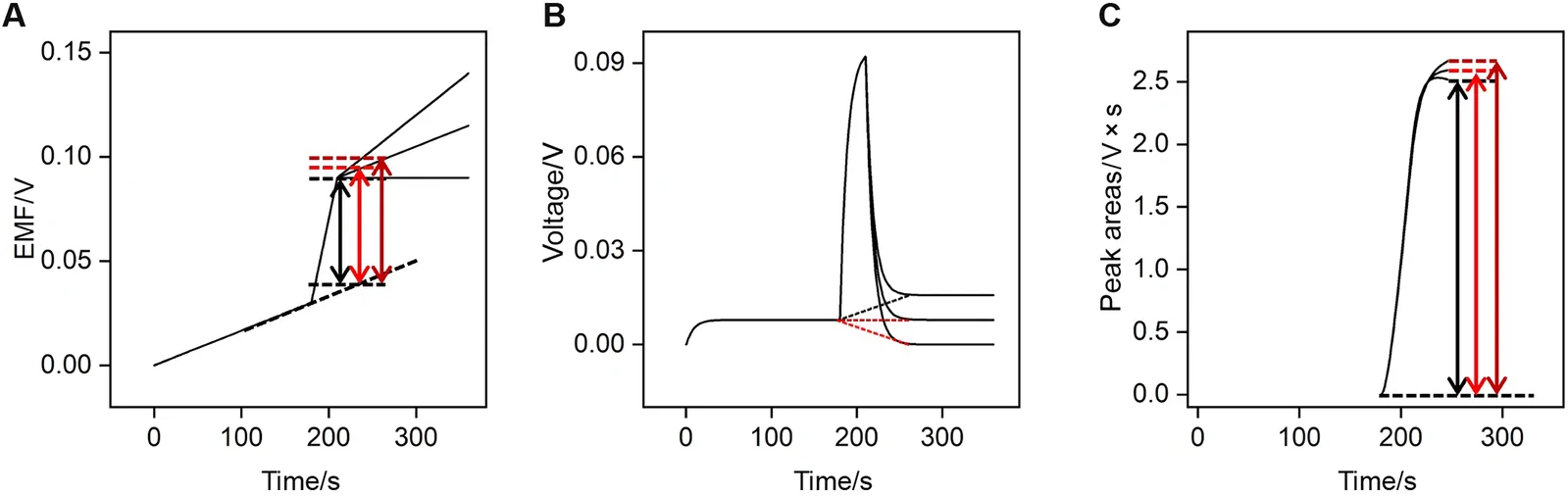
Comparison of potentiometry and pulseometry. (**A**) EMF signals with various drifting rates were assessed by extending the EMF signal prior to sample injection (before 180 s) and measure the EMF change at 240 s. The error may be eliminated effectively only if the drifting rate remains constant. (**B**) Corresponding pulseometry peak signal, choosing the interval from 180 to 240 s for baseline correction. (**C**) Integrated signal for the baseline-corrected pulse. The error introduced from a variable drift is greatly diminished. Peak area converts to ΔEMF with the multiplier *k* = 46.5 s (see Eq. 5).

The rate of EMF drift for unconditioned ISEs may be influenced by multiple factors such as the size and material of the electrodes and the composition of the contacting sample solutions. Thus, these rates may not be predictable. A simplified model mimicking the EMF signals drifting at various rates as depicted in Fig. 3A was used to compare the precision of potentiometry and pulseometry. The initial EMF drift before 180 s was assumed to be identical across all tests. After this, a linearly increasing EMF of 60 mV was applied over a 30-s interval to mimic the injection of sample, after which, however, the EMF in each test was allowed to drift at different rates. As illustrated in Fig. 3A, the signal at 240 s (60 s after the injection) was chosen to obtain the ΔEMF. Only potentials drifting at a constant rate may give the correct ΔEMF value of 60 mV. In most practical cases, this rate will not remain unchanged. An uncertainty will be introduced into the final result and in the case studied here an SD of 4.2 mV was obtained. The corresponding pulseometry signals are presented in Fig. 3B. As we discussed above, the amplitude of the baseline signal is proportional to the rate of EMF drift. Baseline correction was performed for the pulse signal between 180 and 240 s followed by integration over the same time interval Fig. 3C. The standard deviation for three pulseometric measurements in this hypothetical experiment is 1.5 mV, which is an important improvement.

**Fig. 4.**
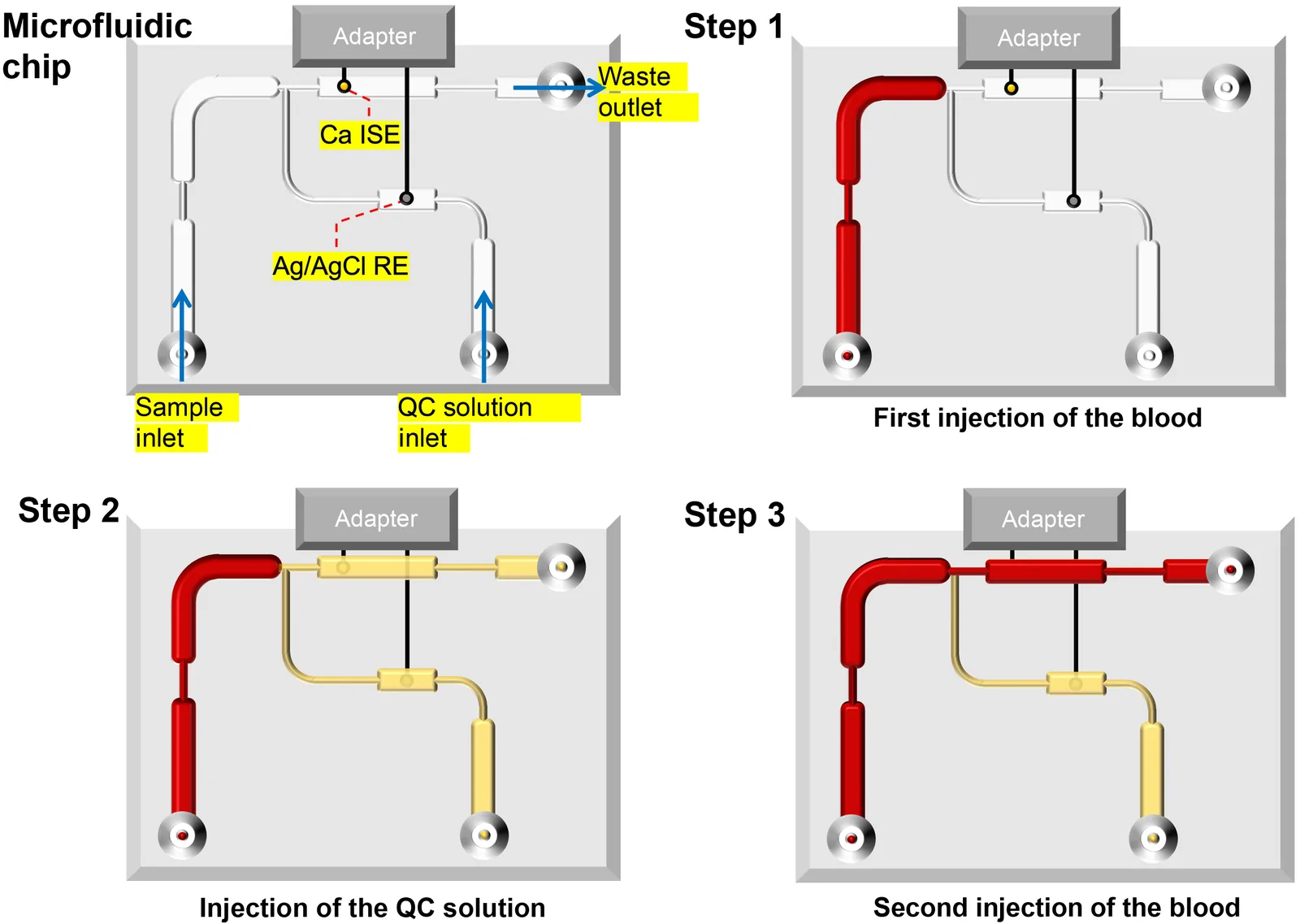
Protocol of the pulseometry measurement. Scheme of the pulseometry procedure with a microfluidic chip for the blood gas analyzer. Blood sample is shown in red; reference QC solution is given in yellow. In the first step, the blood sample is driven to the T-junction in the fluidic cell. The QC solution is then inserted in step 2 to contact the reference element (Ag/AgCl RE) and electrochemical sensor (Ca-ISE). Step 3 displaces the QC solution by the blood sample to perform the final measurement. Note that the reference element remains in contact with the QC solution during this step.

Following the proof of concept established through simplified numerical modeling, the pulseometry method was investigated in collaboration with Eaglenos Sciences with commercially available microfluidic chips used in blood gas electrolyte analyzers. In this chip, a silver/silver chloride (Ag/AgCl) RE is placed in a channel contacting the standard solution only. This channel is connected via a T-shaped junction to the channel containing the indicator electrodes (Fig. 4). In a routine procedure, 150 μl of standard solution is first injected into the chip to fill the channels of ISE and RE, acquiring EMF data for 3 min. Subsequently, a 100 μl of blood sample is injected to the left of the T-junction, displacing both the standard solution and air from the indicator channel, thereby forming an open liquid junction with the reference channel containing standard solution.

The pulseometry protocol required the absence of an air gap between standard and sample measurement. Consequently, the blood sample was initially guided to the T-junction (step 2 in Fig. 4) before injection of standard solution in step 3, followed by the displacement of standard solution by the sample in step 4.

**Fig. 5.**
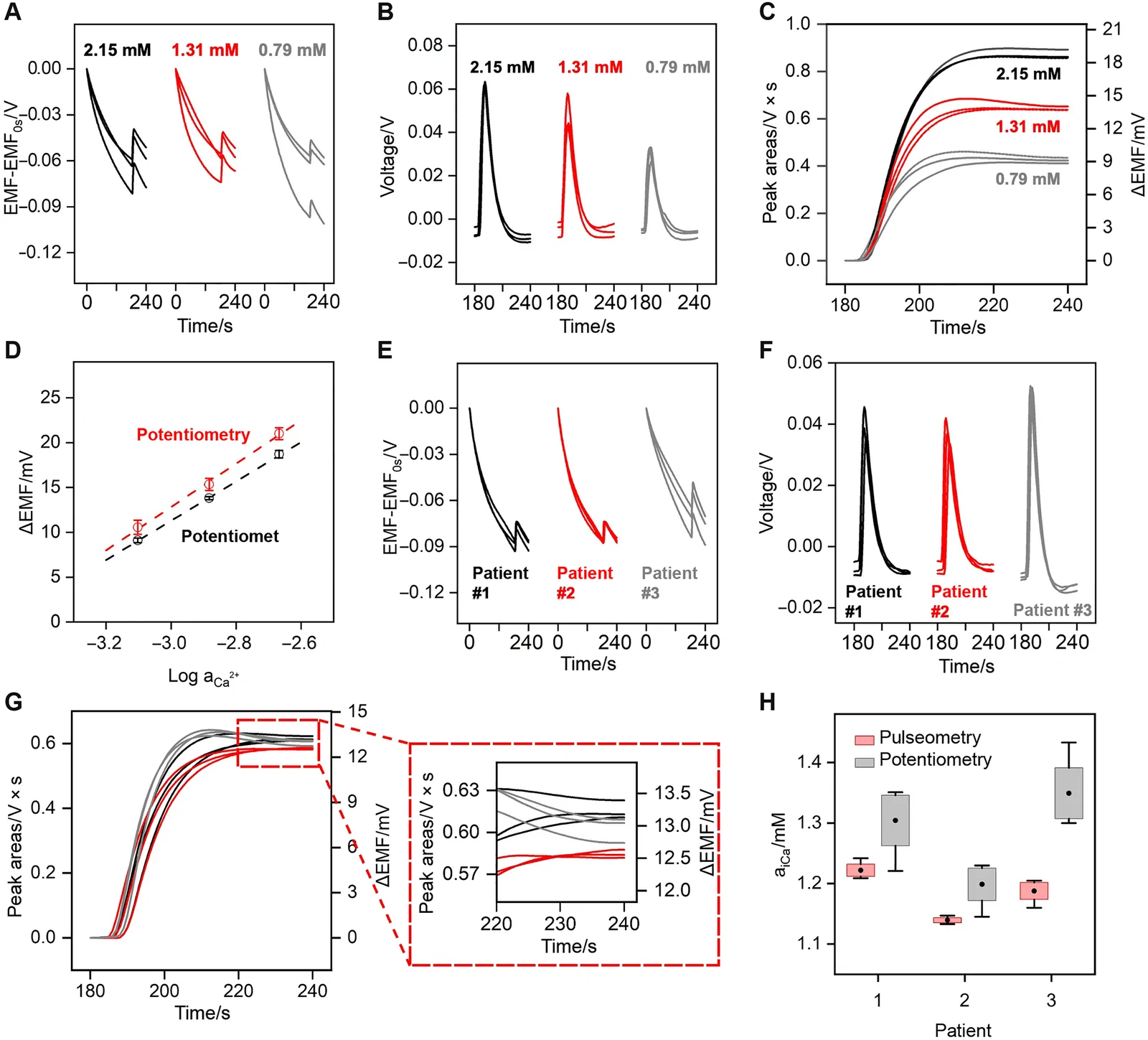
Sensor calibration and whole blood samples assays. (**A**) EMF signals collected potentiometrically for the test card calibration with QC solutions and (**B**) corresponding pulseometry signal for the same experiment. (**C**) Differential voltage signal was integrated with time and converted into the EMF change with eq 5 through *k* = 46.5 s. (**D**) Comparison of the measurements by potentiometry (red dashed line and marker) and pulseometry (black solid line and marker), with greatly diminished error bars for the latter. (**E**) EMF signal collected from potentiometric measurements of iCa level for whole blood samples from three patients and (**F**) corresponding pulseometry signal, showing markedly reduced drift. (**G**) Integrated signal with time and corresponding EMF change for the three blood samples. (**H**) Quantitative data for the blood tests observed by potentiometry and pulseometry, with the latter showing improved precision.

The electrode response slopes of the calcium (Ca) ISEs on the test card were characterized by calibration with quality control (QC) solutions exhibiting Ca levels from 0.79 mM to 2.15 mM (See table S1 for the composition of the QC solutions). This gave a slope of 24.0 mV by potentiometry and a somewhat reduced slope of 22.1 mV by pulseometry using *k* = 46.5 s (fig. S2). The QC solution with Ca concentration of 0.52 mM was used as standard solution for both potentiometry and pulseometry. Each microfluidic chip was used for a single measurement with the two methods before being discarded. The standard deviation of the EMF change for three calibration solutions found by the pulseometry was 0.15, 0.38, and 0.27 mV. These values were 36, 74, and 67% smaller than those found by potentiometry (Fig. 5, A to D).

The healthy level of ionized calcium (iCa) in human plasma ranges from about 1.1 to 1.29 mM. Blood samples collected from three different volunteers were measured as a real-world practical application. Each patient was tested with three new test cards each by both potentiometry and pulseometry. Because both potentiometric and pulseometric measurements were repeated three times (n=3), the SD (σ) was used to evaluate measurement precision instead of the SE ($\text{SE}=\sigma /\sqrt{n}$). In future clinical investigations involving a large number of samples independently measured by the pulseometry and potentiometry, SE would be more appropriate for evaluating analytical precision. The iCa level found from three samples were 1.22 ± 0.018, 1.14 ± 0.008, and 1.19 ± 0.024 mM with pulseometry and 1.30 ± 0.072, 1.10 ± 0.046, and 1.35 ± 0.073 mM with potentiometry.

To evaluate the accuracy of pulseometry for blood ion analysis, the measured results were compared with reference values obtained from a commercial blood gas analyzer manufactured by Radiometer, which is widely used in clinical laboratories for routine blood electrolyte measurements (*40*, *41*). Identical blood samples were analyzed using both pulseometry and conventional potentiometry under the same experimental conditions, and the obtained values were subsequently compared with the reference measurements provided by the Radiometer analyzer. As summarized in Table 1, the ion concentrations determined by pulseometry were consistently closer to those of the reference method than the values obtained by conventional potentiometry. While these results are from a limited dataset, they indicate that pulseometry may provide enhanced analytical accuracy for blood ion measurements and may reduce the systematic deviation observed in conventional potentiometric measurements.

**Table 1.** iCa level of the blood collected form three patients measured by a commercial device and with our microfluidic chip by two potentiometry and pulseometry.

Patient#	1	2	3
	Average/mM	Average/mM	Average/mM
Radiometer ABL 90	1.21	1.17	1.23
Pulseometry	1.22 ± 0.018	1.14 ± 0.008	1.19 ± 0.024
Potentiometry	1.30 ± 0.072	1.20 ± 0.046	1.35 ± 0.073

**Fig. 6.**
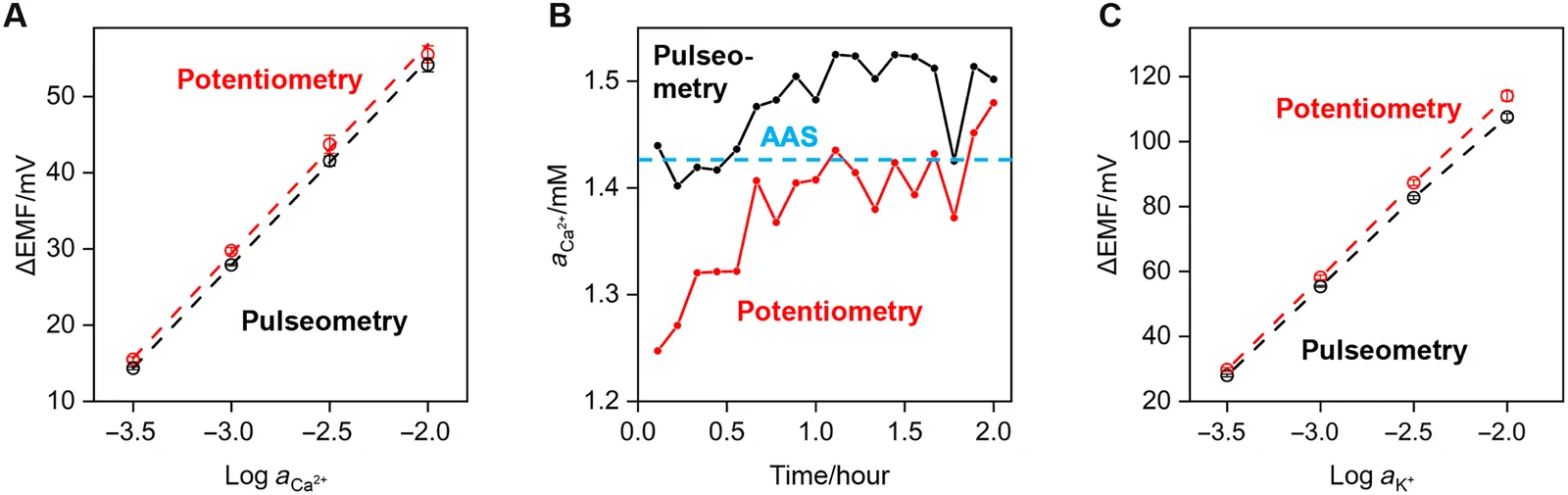
Evaluation of the pulseometry with conventional solid contact ion selective electrodes. (**A**) Solid contacted Ca-ISE was calibrated with the potentiometry and pulseometry. (**B**) Ca concentration was measured by the ISE through pulseometry and potentiometry, and a smaller signal drift was found for pulseometry. (**C**) K-ISE calibrated by pulseometry and potentiometry.

Pulseometry was also evaluated using unconditioned Ca-ISEs under continuous measurement conditions. Four Ca-ISEs incorporating glassy carbon electrodes were freshly prepared and used without conditioning. The Nernstian responses of the electrodes were calibrated using three Ca-ISEs, yielding slopes of 26.8 mV per decade for conventional potentiometry and 26.6 mV per decade for pulseometry. In addition, the standard deviations obtained from pulseometric calibration at four different Ca^2+^ activities were 43, 71, 55, and 19% lower, respectively, than those obtained by potentiometry (see Fig. 6A), indicating improved measurement precision with the pulseometric approach. A fourth unconditioned Ca-ISE was subsequently used for continuous monitoring of total Ca^2+^ activity in an acidified bottled water sample. The total Ca concentration of this sample was determined to be 1.42 mM by atomic absorption spectroscopy. Owing to the absence of electrode preconditioning, signal drift was expected during the electrochemical measurements. As shown in Fig. 6B, over a 2-hour measurement period, the pulseometric response varied from 1.40 to 1.52 mM, whereas the potentiometric response changed from 1.24 to 1.48 mM. Linear regression analysis further revealed that the drift of pulseometry corresponded to 0.052 mM hour^−1^, which is substantially lower than the 0.096 mM hour^−1^ observed for the potentiometry. These results demonstrate that pulseometry not only improves precision in single use measurements in the clinical assay but also provides enhanced stability for continuous monitoring applications involving sensors susceptible to signal drift.

The method is, in principle, compatible with any potentiometric probe without requiring modifications to the existing experimental setup. To further evaluate its compatibility with other potentiometric sensors, pulseometry experiments were carried out using K-ISEs. Three K-ISEs using sodium tetrakis[3,5-bis(trifluoromethyl)phenyl]borate (NaTFPB) as the ion exchanger were prepared according to the procedures described in Materials and Methods. A slightly lower Nernstian slope of 53.2 mV was obtained with pulseometry compared with 56.4 mV measured by conventional potentiometry. The SDs obtained from three pulseometric measurements in KCl solutions with four different ion activities were found to be 29 to 65% smaller than those obtained using potentiometric measurements, again indicating improved measurement precision with the pulseometric approach (Fig. 6C).

## DISCUSSION

In this study, we developed a readout strategy, termed pulseometry, as an alternative to conventional potentiometry for potentiometric sensors. When evaluated using a commercial blood gas analyzer test card, pulseometry demonstrated markedly improved precision in measuring calcium levels of whole blood samples from three patients, yielding 67 to 84% improved SDs than traditional potentiometry. Furthermore, the pulseometry results were closer to those obtained with a commercial analyzer used as a reference method, suggesting also improved accuracy.

This work introduces a methodology-driven pathway to enhance the precision of POCT devices and offers a practical route toward improved instrumentation. Although the present study used manual sample injection in an environment susceptible to electromagnetic noise, further gains in precision are anticipated with automated operation and electromagnetic shielding in future device integration. In the current experimental setup, air bubbles introduced during blood injection may temporarily open-circuit the ion-selective and reference electrodes, resulting in undesired potential fluctuations. Therefore, compared with conventional potentiometric measurements, an additional sample injection step was required in pulseometry experiments to purge residual gas from the microfluidic chip and avoid sudden potential jumps. This additional step may be eliminated in future implementations by maintaining the cell potential with a digital-to-analog converter during sample injection. Furthermore, a simple linear baseline subtraction was applied in this manuscript to correct the potential drift. For further improvement of the precision, a more complex model for the subtraction of a changing baselines when observing time-variable drifts may result in even better precision.

## MATERIALS AND METHODS

### Reagents

Sodium chloride (NaCl, ≥99.0%), potassium chloride (KCl, ≥99.0%), calcium chloride (CaCl_2_; ≥99.0%), hydrochloric acid (HCl, quality level of 300) were used to prepare the standard and the sample solutions. Calcium ionophore IV (*N*,*N*-dicyclohexyl-*N*′,*N*′-dioctadecyl-3-oxapentanediamide, ≥98.0%), potassium ionophore I (Valinomycin, ≥99.0%), NaTFPB (quality level of 100), tetradodecylammonium tetrakis(4-chlorophenyl)borate (ETH 500, quality level of 200), poly(vinyl chloride) (PVC; high molecule weight, quality level of 200), 2-nitrophenyloctylether (NPOE; ≥99.0%), tetrahydrofuran (THF; ≥99.0%) were used to prepare the ion sensing cocktails for the K and Ca-ISE. All reagents were sourced from Sigma-Aldrich.

### Equipment

Metallized polyester capacitors (10 μF), resistors, strip board, AD 820 operational amplifier, and INA 114 instrumentation amplifier were sourced from digikey. A 5-V dc power supply purchased from RND Lab. Potential signals were measured with EMF16 data acquisition system from Lawson Laboratories. Quality control solutions of different Ca levels and microfluidic chips were sourced from Eaglenos Sciences. Left-over blood sample from three unidentified (anonymous) subjects collected in clinical settings was used for our experiments. All the blood sample were collected and stored in sodium heparin tubes and kept in the fridge (4°C). The blood samples and QC solutions were delivered into the test card with 1-ml plastic syringes. For the flow cell experiment, glassy carbon electrodes were purchased from Oesch Sensor Technology. Ag/AgCl reference electrode with two liquid junctions was sourced from Metrohm. The flow cell was manufactured by the machine shop of our department. A peristaltic pump and a switching valve from Runze Fluidic were used to deliver the solutions to the flow cell.

### Instrumentation of pulseometry analyzer

The electronic components were implanted on the strip board according to the circuit shown in the fig. S1. A 5-V voltage sourced from the dc power supplier was converted into ±5 V with a booster converter to power all the amplifiers on the board. A picture of this board is illustrated in fig. S3A. Each electrode on the test card was attached to a female BNC (Bayonet Neill–Concelman) connector. The ISE was connected to the input of the board with BNC cables. The potentiometry signal was collected at the input of the board, and the pulseometry signal was collected simultaneously from the output of the board with EMF 16 voltmeter and computer (fig. S3, B and C).

### Ca measurement with microfluidic chips

A 50 μl of the blood sample was initially injected into the microfluidic chip with syringe, followed by the injection of 150 μl of reference QC solution. The second injection of 50 μl of blood was done 180 s after the QC solution injection. The EMF at 210 and 240 s after sample injection was chosen to form the baseline. We may find the EMF jump before and after the sample injection by extending this baseline and comparing it to the EMF value at 0 s. To obtain the pulseometry result, the potential at 180 and 240 s was used to form the baseline. The voltage signal over this time period was integrated to give the signal change.

### Ca measurement with commercialized analyzers

The same blood samples sourced from three volunteers were measured with the ABL 90 blood gas analyzer from Radiometer. About 100 μl of the blood sample was drawn from the tube with a syringe for the analyzer, while the remaining assay was carried out automatically by the instrument. The measurement was carried out once for each sample and used as the reference value.

### Preparation of the ion sensing component cocktail for different ISEs

For K-ISE, 3.3 mg of valinomycin, 0.9 mg of NaTFPB, 20 mg of ETH 500, 60 mg of PVC, 120 mg of NPOE were dissolved in 2 ml of THF. For Ca-ISE, 2.4 mg of Ca ionophore IV, 0.9 mg of NaTFPB, 20 mg of ETH 500, 60 mg of PVC, 120 mg of NPOE were dissolved in 2 mL of THF.

### Preparation of the ion selective electrodes

The glassy carbon electrodes were polished with diamond spray with different sizes (from 6 to 0.25 μm) and then cleaned by ultrasonicating in the mixture of water and ethanol (1:1). Three aliquots of 15 μl of carbon nanotube dispersion in THF (1 mg/ml) were drop--cast on top of the glassy carbon surface with the help of a customed made mask. These masks were made by stacking 10 layers of scotch tape on each other. A hole of 3.5 mm was punched on the mask to expose the glassy carbon surface in the air. The mask was then removed. A 50 μl of ion sensing component cocktail was cast to cover the whole electrode surface. The cocktail was cast three times after the previous drop dried.

### Electrolyte solutions preparation for the flow cell experiment

The standard solutions of the potassium were prepared by diluting the 1 M KCl stock solution with the 0.1 M NaCl. The background 0.1 M NaCl solution is essential to provide the conductivity to the solution in the thin flow cell channel.

Standard solutions of calcium were prepared by diluting a 1 M CaCl_2_ stock solution with 0.1 M KCl and 0.1 M HCl. The acidified solutions keep the consistency of the Ca concentration during the experiment.

The bottled water sample was prepared by mixing 89% (v/v) bottled water, 10% (v/v) 1 M KCl, and 1% (v/v) 1 M HCl.

### Ca and K measurement with flow cell and solid contact ISE

A potentiometric cell was constructed by the ISEs and the double liquid junction Ag/AgCl reference electrode in a flow cell as illustrated in the fig. S5A. All the standard and sample solutions were prepared as indicated and stored in the plastic bottles. The solutions were delivered into the flow cell as shown in fig. S5B by a peristaltic pump at the rate of 2 ml/min. The Ag/AgCl RE was connected to the ground of the pulseometry circuit board, and the ISE was connected to the input of the voltage follower. The pulseometry signal was collected simultaneously from the output of the board with EMF 16 voltmeter and computer.

## Supplementary Materials

### This PDF file includes:

Figs. S1 to S7 Table S1 Mathematica code for data analysis

## References

[1] C. P. Price, Point of care testing. BMJ 322, 1285–1288 (2001). 11375233 10.1136/bmj.322.7297.1285 PMC1120384

[2] R.-J. Yang, L.-M. Fu, H.-H. Hou, Review and perspectives on microfluidic flow cytometers. Sens. Actuators B Chem. 266, 26–45 (2018).

[3] S. Stavrakis, G. Holzner, J. Choo, A. DeMello, High-throughput microfluidic imaging flow cytometry. Curr. Opin. Biotechnol. 55, 36–43 (2019). 30118968 10.1016/j.copbio.2018.08.002

[4] A. Bange, H. B. Halsall, W. R. Heineman, Microfluidic immunosensor systems. Biosens. Bioelectron. 20, 2488–2503 (2005). 15854821 10.1016/j.bios.2004.10.016

[5] L. Mou, X. Jiang, Materials for microfluidic immunoassays: A review. Adv. Healthc. Mater. 6, 1601403 (2017). 10.1002/adhm.201601403 28322517

[6] H. Jiang, X. Weng, D. Li, Microfluidic whole-blood immunoassays. Microfluid. Nanofluid. 10, 941–964 (2011).

[7] S. Mross, S. Pierrat, T. Zimmermann, M. Kraft, Microfluidic enzymatic biosensing systems: A review. Biosens. Bioelectron. 70, 376–391 (2015). 25841121 10.1016/j.bios.2015.03.049

[8] H. Yamaguchi, M. Miyazaki, Enzyme-immobilized microfluidic devices for biomolecule detection. Trends Anal. Chem. 159, 116908 (2023).

[9] Z. Slouka, S. Senapati, H.-C. Chang, Microfluidic systems with ion-selective membranes. Annu. Rev. Anal. 7, 317–335 (2014). 10.1146/annurev-anchem-071213-020155 24818814

[10] S. Chen, Z. Qiao, Y. Niu, J. C. Yeo, Y. Liu, J. Qi, S. Fan, X. Liu, J. Y. Lee, C. T. Lim, Wearable flexible microfluidic sensing technologies. Nat. Rev. Bioeng. 1, 950–971 (2023).

[11] H. Tabasum, N. Gill, R. Mishra, S. Lone, Wearable microfluidic-based e-skin sweat sensors. RSC Adv. 12, 8691–8707 (2022). 35424805 10.1039/d1ra07888g PMC8985157

[12] G. Lisak, J. Cui, J. Bobacka, Based microfluidic sampling for potentiometric determination of ions. Sens. Actuators B Chem. 207, 933–939 (2015).

[13] C. Lou, H. Yang, Y. Hou, H. Huang, J. Qiu, C. Wang, Y. Sang, H. Liu, L. Han, Microfluidic platforms for real-time in situ monitoring of biomarkers for cellular processes. Adv. Mater. 36, e2307051 (2024). 37844125 10.1002/adma.202307051

[14] O. G. Chavez-Pineda, R. Rodriguez-Moncayo, D. F. Cedillo-Alcantar, P. E. Guevara-Pantoja, J. U. Amador-Hernandez, J. L. Garcia-Cordero, Microfluidic systems for the analysis of blood-derived molecular biomarkers. Electrophoresis 43, 1667–1700 (2022). 35767850 10.1002/elps.202200067

[15] W. Heng, S. Yin, J. Min, C. Wang, H. Han, E. Shirzaei Sani, J. Li, Y. Song, H. B. Rossiter, W. Gao, A smart mask for exhaled breath condensate harvesting and analysis. Science 385, 954–961 (2024). 39208112 10.1126/science.adn6471 PMC12168143

[16] W. Gao, S. Emaminejad, H. Y. Y. Nyein, S. Challa, K. Chen, A. Peck, H. M. Fahad, H. Ota, H. Shiraki, D. Kiriya, Fully integrated wearable sensor arrays for multiplexed in situ perspiration analysis. Nature 529, 509–514 (2016). 26819044 10.1038/nature16521 PMC4996079

[17] M. Wang, Y. Yang, J. Min, Y. Song, J. Tu, D. Mukasa, C. Ye, C. Xu, N. Heflin, J. S. McCune, A wearable electrochemical biosensor for the monitoring of metabolites and nutrients. Nat. Biomed. Eng. 6, 1225–1235 (2022). 35970928 10.1038/s41551-022-00916-z PMC10432133

[18] A. L. Gonzalez, L. S. Waddell, Blood gas analyzers. Top. Companion Anim. Med. 31, 27–34 (2016). 27451046 10.1053/j.tcam.2016.05.001

[19] C.-C. Lee, S.-Y. Lu, C.-X. Yu, C.-C. Tseng, K.-H. Huang, L.-M. Fu, Electrochemical biosensor microchip platform for rapid whole-blood chloride ions quantification in CKD point-of-care monitoring. Talanta 302, 129320 (2026). 41520402 10.1016/j.talanta.2025.129320

[20] Y. Yuan, S. Feng, M. E. E. Alahi, A. Nag, N. Afsarimanesh, H. Zhang, S. He, Development of an Internet of Things based electrochemical microfluidic system for free calcium detection. Appl. Sci. 8, 1357 (2018).

[21] S. Farahani, D. L. Glasco, M. M. Elhassan, P. Sireesha, J. G. Bell, Integration of 3D printed Mg 2+ potentiometric sensors into microfluidic devices for bioanalysis. Lab Chip 24, 4096–4104 (2024). 39086302 10.1039/d4lc00407h

[22] C.-C. Tseng, S.-Y. Lu, S.-J. Chen, J.-M. Wang, L.-M. Fu, Y.-H. Wu, Microfluidic aptasensor POC device for determination of whole blood potassium. Anal. Chim. Acta 1203, 339722 (2022). 35361435 10.1016/j.aca.2022.339722

[23] J.-H. Jin, J. H. Kim, S. K. Lee, S. J. Choi, C. W. Park, N. K. Min, A fully integrated paper-microfluidic electrochemical device for simultaneous analysis of physiologic blood ions. Sensors 18, 104 (2018). 29301270 10.3390/s18010104 PMC5796313

[24] P. Zhao, W.-J. Cai, An improved potentiometric *p*CO_2_ microelectrode. Anal. Chem. 69, 5052–5058 (1997).

[25] E. Bakker, P. Bühlmann, E. Pretsch, Carrier-based ion-selective electrodes and bulk optodes. 1. General characteristics. Chem. Rev. 97, 3083–3132 (1997). 11851486 10.1021/cr940394a

[26] M. Guzinski, J. M. Jarvis, B. D. Pendley, E. Lindner, Equilibration time of solid contact ion-selective electrodes. Anal. Chem. 87, 6654–6659 (2015). 26005770 10.1021/acs.analchem.5b00775

[27] E. Bakker, Selectivity of liquid membrane ion-selective electrodes. Electroanalysis 9, 7–12 (1997).

[28] J.-P. Veder, R. De Marco, G. Clarke, R. Chester, A. Nelson, K. Prince, E. Pretsch, E. Bakker, Elimination of undesirable water layers in solid-contact polymeric ion-selective electrodes. Anal. Chem. 80, 6731–6740 (2008). 18671410 10.1021/ac800823f PMC2628482

[29] B. Hambly, M. Guzinski, B. Pendley, E. Lindner, Evaluation, pitfalls and recommendations for the “water layer test” for solid contact ion-selective electrodes. Electroanalysis 32, 781–791 (2020).

[30] S. N. Cozzette, G. Davis, L. A. Holleritter, I. R. Lauks, S. Piznik, N. J. Smit, J. A. Tirinato, H. J. Wieck, M. P. Zelin, Method for analytically utilizing microfabricated sensors during wet-up. U.S. Patent US5112455A, 12 May 1992.

[31] T. Han, U. Mattinen, J. Bobacka, Improving the sensitivity of solid-contact ion-selective electrodes by using coulometric signal transduction. ACS Sens. 4, 900–906 (2019). 30844250 10.1021/acssensors.8b01649 PMC6727616

[32] Z. Jarolimova, T. Han, U. Mattinen, J. Bobacka, E. Bakker, Capacitive model for coulometric readout of ion-selective electrodes. Anal. Chem. 90, 8700–8707 (2018). 29906094 10.1021/acs.analchem.8b02145

[33] U. Vanamo, E. Hupa, V. Yrjänä, J. Bobacka, New signal readout principle for solid-contact ion-selective electrodes. Anal. Chem. 88, 4369–4374 (2016). 27018524 10.1021/acs.analchem.5b04800

[34] P. Kraikaew, S. Jeanneret, Y. Soda, T. Cherubini, E. Bakker, Ultrasensitive seawater pH measurement by capacitive readout of potentiometric sensors. ACS Sens. 5, 650–654 (2020). 32106675 10.1021/acssensors.0c00031

[35] R. Nussbaum, S. Jeanneret, E. Bakker, Increasing the sensitivity of pH glass electrodes with constant potential coulometry at zero current. Anal. Chem. 96, 6436–6443 (2024). 38593052 10.1021/acs.analchem.4c00592 PMC11044110

[36] P. Kraikaew, Y. Soda, R. Nussbaum, S. Jeanneret, E. Bakker, Portable instrument and current polarization limitations of high sensitivity constant-potential capacitive readout with polymeric ion-selective membranes. Sens. Actuator B Chem. 379, 133220 (2023).

[37] P. Kraikaew, S. K. Sailapu, E. Bakker, Electronic control of constant potential capacitive readout of ion-selective electrodes for high precision sensing. Sens. Actuator B Chem. 344, 130282 (2021).

[38] Y. Wu, Y. Zhang, A. Qileng, E. Bakker, Self-powered potentiometric sensor with relational operation function to capture concentration excursions. Anal. Chem. 96, 18401–18407 (2024). 39523720 10.1021/acs.analchem.4c03081

[39] Y. Wu, A. Qileng, E. Bakker, Self-powered optical ion sensor array based on potentiometric probes coupled to electronic paper. Sens. Actuator B Chem. 396, 134561 (2023).

[40] A. Y. Al-Shahrani, J. Khan, Evaluation of ABL90 and ABL800 radiometer blood gas analyzers: Challenges and applications in point-of-care cancer diagnostics in Saudi Arabia. Healthcare 13, 331 (2025). 39942520 10.3390/healthcare13030331 PMC11817300

[41] M. E. García-Payá, M. Fernández-González, N. V. Morote, Á. B. Cobos, M. C. T. Díaz, J. F. S. Hernández, Performance of the radiometer ABL-90 FLEX blood gas analyzer compared with similar analyzers. Lab. Med. 44, e52–e61 (2013).

