# Supplementary material for: Pulseometry: A differential readout to compensate signal drift of potentiometric probes
Source: Sci Adv. 2026 Aug 21;12(34):eaef5993. doi: 10.1126/sciadv.aef5993 (PMC13496203; doi:10.1126/sciadv.aef5993)
Supplement: Supplementary file 1 [file sciadv.aef5993_sm.pdf]

Science Advances
AAAS

Supplementary Materials for

Pulseometry: A differential readout to compensate signal drift of potentiometric probes

Yaotian Wu *et al.*

Corresponding author: Eric Bakker, eric.bakker@unige.ch

Sci. Adv. **12**, eaef5993 (2026)
DOI: 10.1126/sciadv.aef5993

This PDF file includes:

Figs. S1 to S7
Table S1
Mathematica code for data analysis

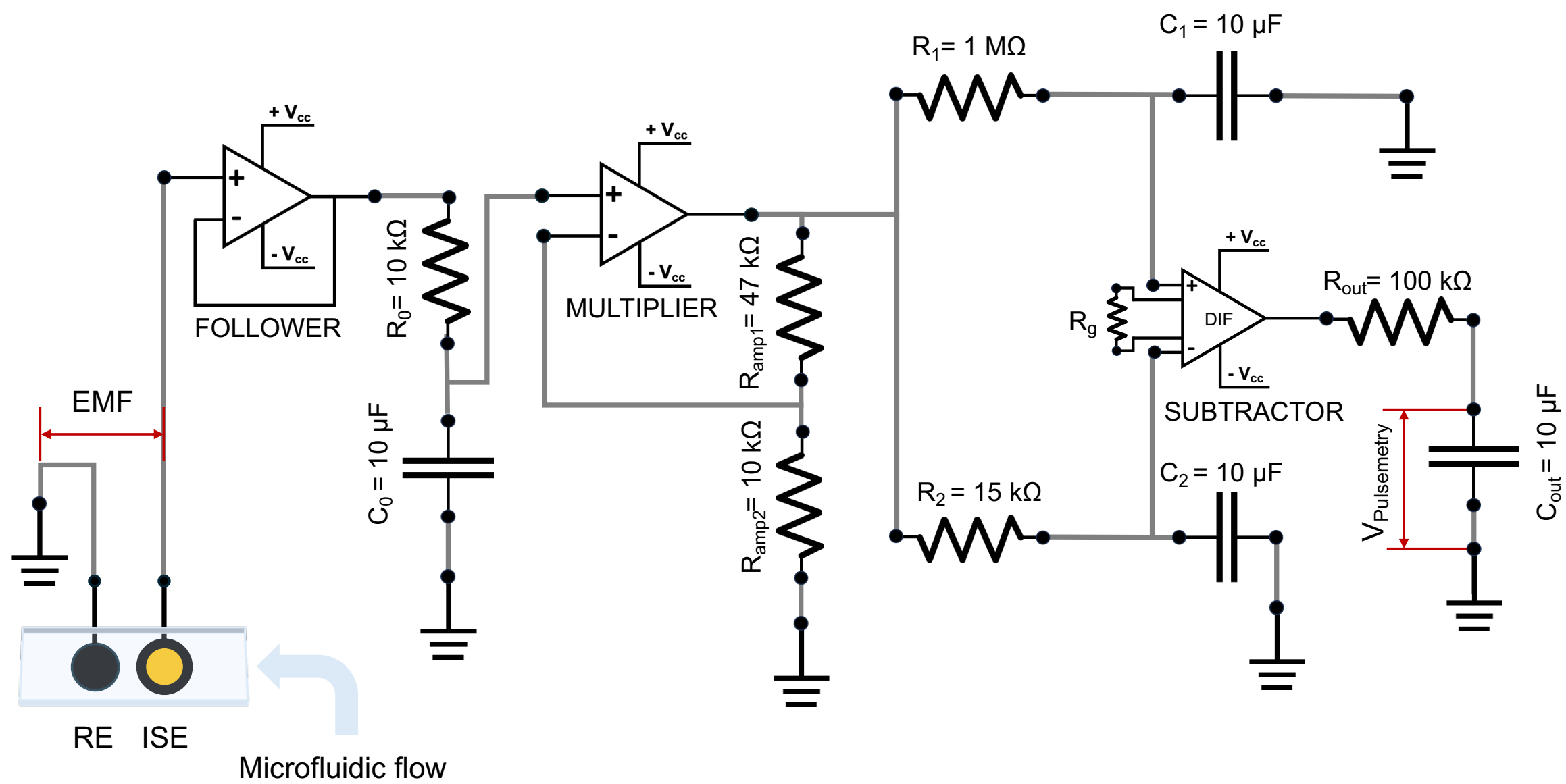

Fig. S1. Scheme of the circuit. The open circuit voltage was measured with a voltage follower and then filtered and amplified. The amplified signal charges two parallel connected RC loop with different time constants. The voltage difference is measured with a subtractor. The output signal is filtered again to avoid high frequency electronic noise.

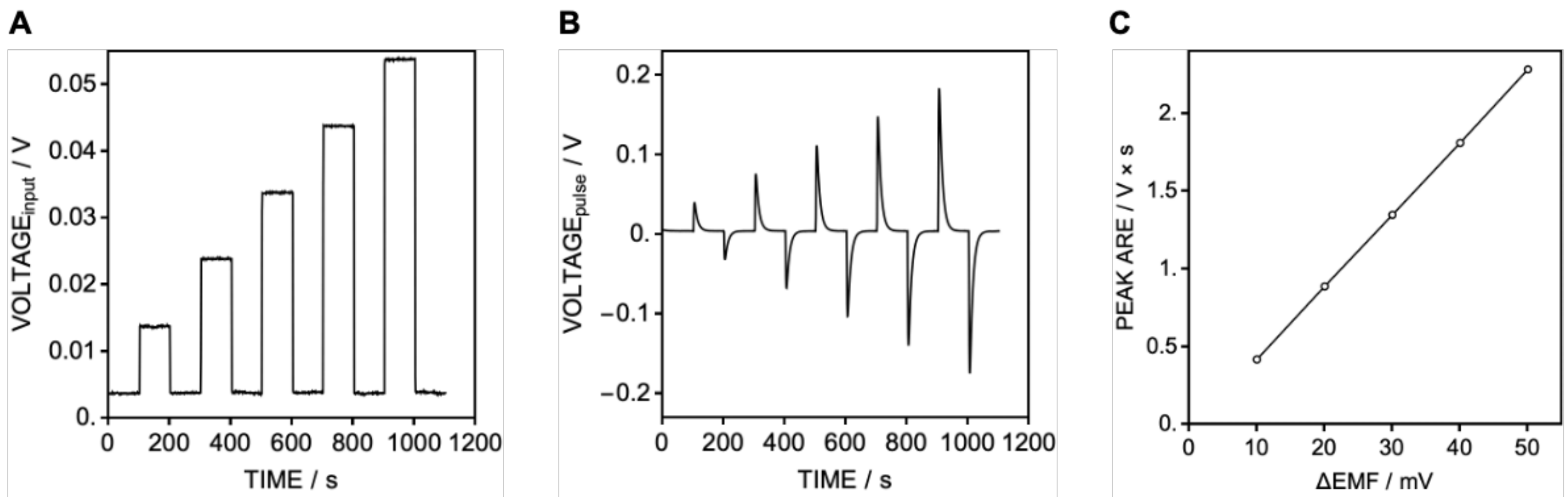

Fig. S2. Calibration of the pulseometry circuit. A stimulation voltage shown in figure (A) is applied to the circuit to mimic the EMF change brought by the sample injection. (B) pulse signals are generated by the pulseometry circuit. (C) The peak areas of these pulses are found to be proportional to the amplitudes of the voltage changes in A.

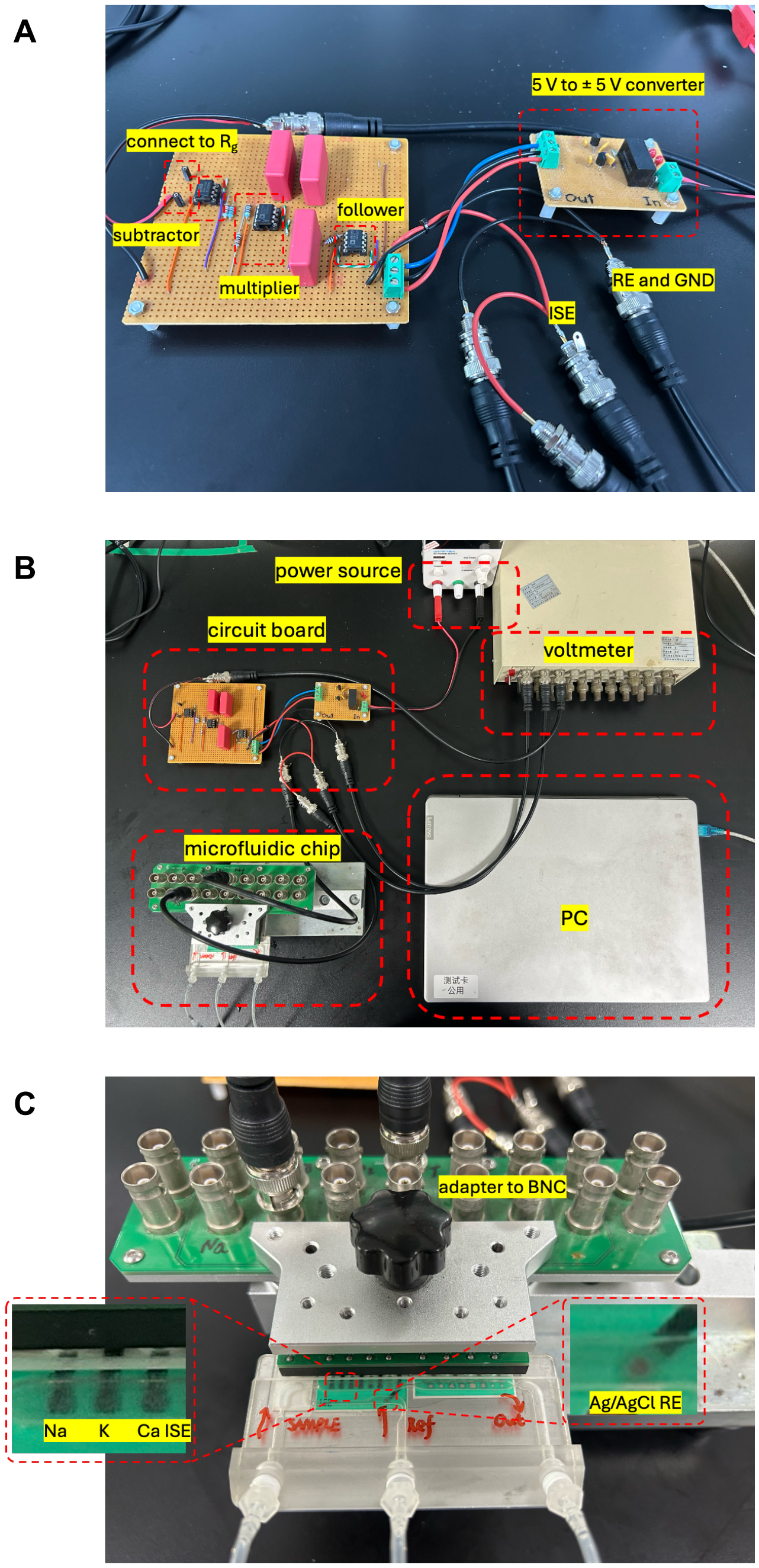

Fig. S3. Photographs of the pulsemetry circuit. (A) The amplifiers are powered by the converter. The RE is connected to the ground of our circuit. The voltage of the ISE against the RE is used for direct potentiometry readout and stimulation of the pulseometry. The output of the subtractor is used as the signal for the pulseometry. (B) The macro-ISEs on the microfluidic chip is connected to the board with a marco-electrode-BNC adapter. The signal is measured by the voltmeter and stored in the PC. (C) Multiple different ISEs and one Ag/AgCl RE are printed on the test card. In this work, only the Ca-ISE is used. The signal is transferred to the pulseometry board through a cabled BNC adapter.

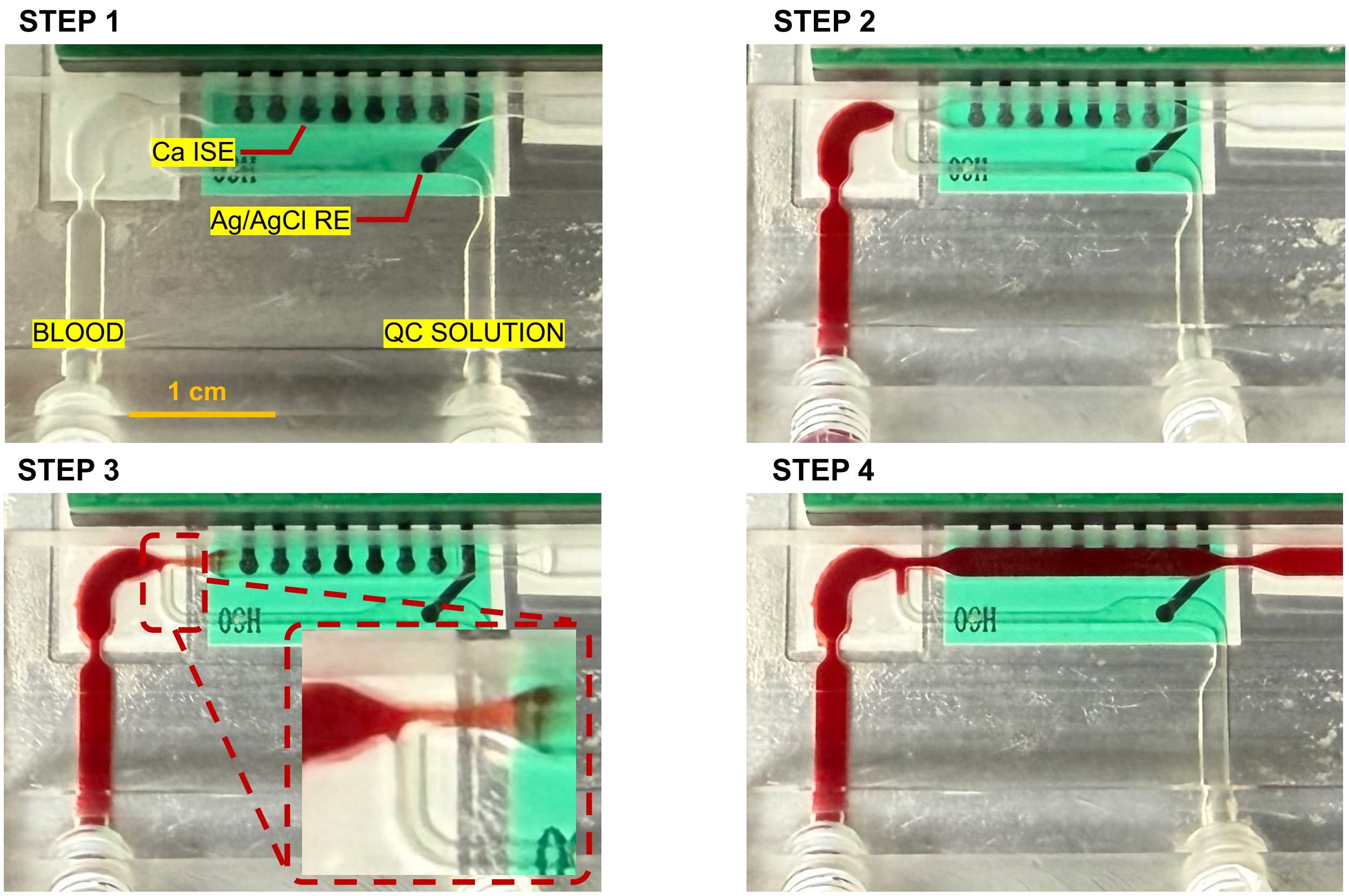

Fig. S4. Photographs of the blood test card during pulseometric Ca measurement. The blood sample is first injected to fill the channel just left to the T-junction (Step 2). A QC solution serving as reference solution is then injected to purge residual air in the microfluidic chip (Step 3). The blood sample is then injected to displace the QC solution at 180 s (Step 4). The sensor voltage changing from reference to sample solution is then measured.

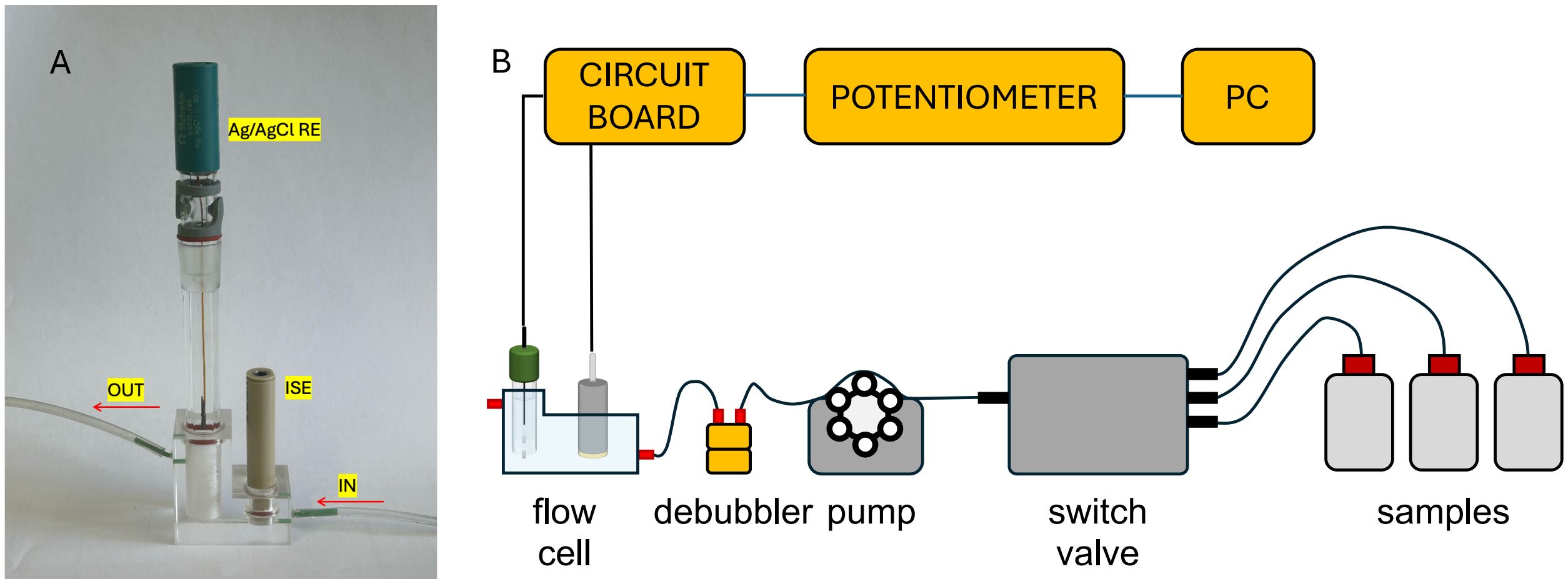

Fig. S5. (A) The photograph of the potentiometric cell composed by the flow cell and the electrodes and (B) the scheme of the experiment setup. The sample and the standards are injected from the right to the left with a peristaltic pump.

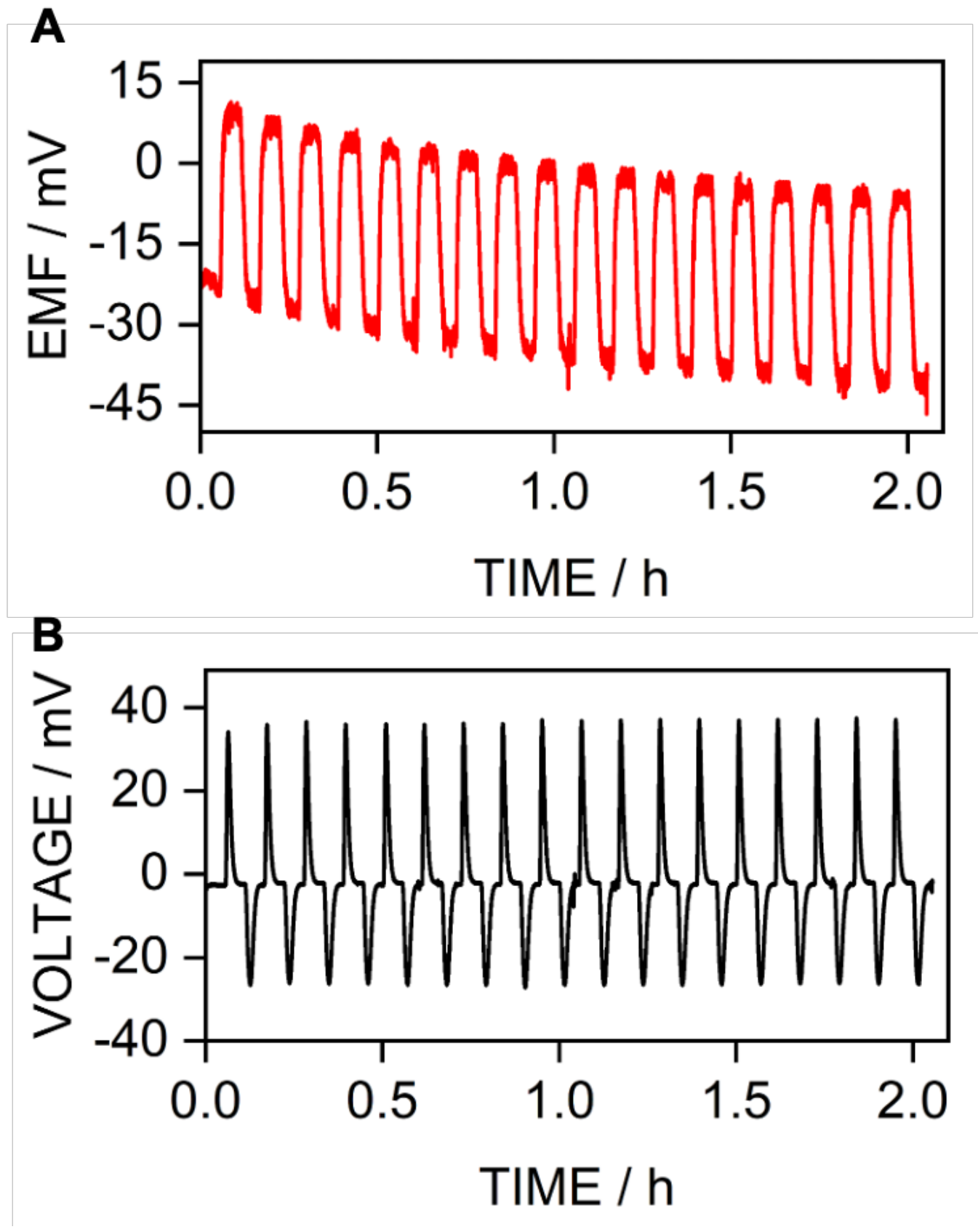

Fig. S6. Signal time-trace for the continuous Ca level monitoring of a bottled water sample. (A) potentiometry and (B) potentiometry experiment of the Ca concentration in the bottled water.

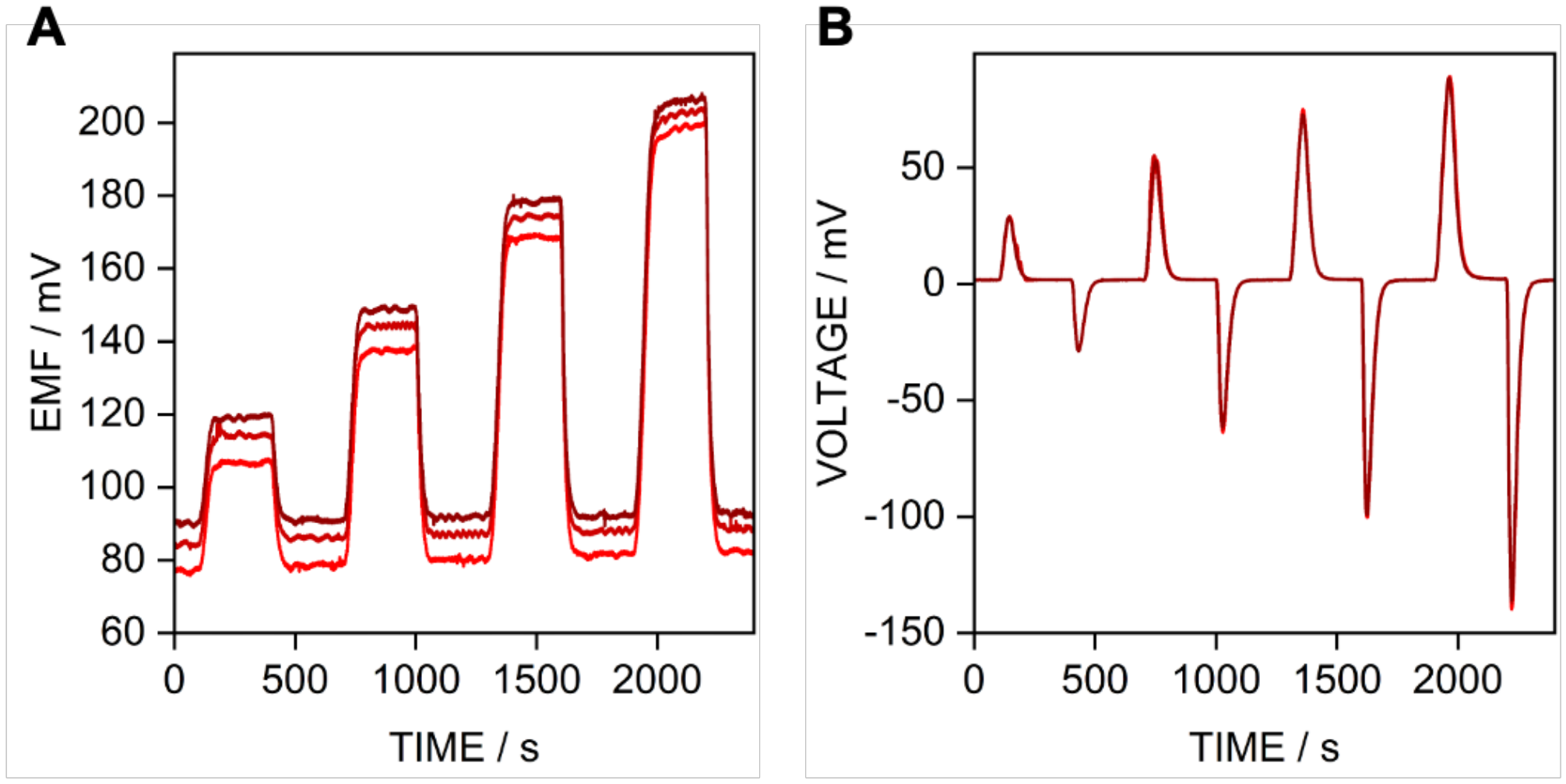

Fig. S7. Calibration of an unconditioned K-ISE. The K-ISE is calibrated with solutions containing 10^{-4} M to 10^{-2} M KCl by (A) potentiometry and (B) pulseometry.

Table S1. Composition of the quality control solutions

	Level 1	Level 2	Level 3	Reference
pH	7.223	7.422	7.637	7.523
pCO2	67.6	47.71	24.8	13.1
pO2	77.7	125.33	167.1	219
Na	121.7 mM	140.84 mM	168.4 mM	181.9 mM
K	1.81 mM	4.01 mM	6.85 mM	9 mM
Cl	83.8 mM	96.15mM	119.1mM	147.6 mM
Ca	2.15 mM	1.31 mM	0.79 mM	0.52 mM
Mg	0.88 mM	0.60 mM	0.39 mM	0.16 mM
Glucose	2.70 mM	6.54 mM	15.64 mM	3.63 mM
Lactate	6.40 mM	3.85 mM	1.00 mM	1 mM

Mathematica code for data analysis
For potentiometry:

The data was first imported from the excel sheets as a list of values containing time and potential, formatted as {time, voltage} using the following command:
data = Import[FilePath][[1]];
*(*Import the original data from the Excel sheets as a list of {time, voltage}*)*

The EMF value at the 150th and 180th seconds was used as baseline for correcting the potential drift during the measurement. This baseline was extended to the 210th second and the EMF change was then calculated by subtraction as follows:

slopebaseline = (data[[180, 2]] - data[[150, 2]])/30;
*(*Find the slope of the baseline*)*
emf210sbaseline = data[[180, 2]] + slopebaseline*30;
*(*extended baseline value is calculated*)*
deltaemf = data[[210, 2]] - emf210sbaseline
*(*the EMF change was calculated as the EMF value subtracted by the baseline value*)*

For Pulseometry:
The data was first imported from the excel sheet as a list of {time, voltage} also. With the following command:
data = Import[FilePath][[1]];
*(*Import the original data from the Excel as a listr of {time,voltage}*)*

The line connecting the EMF values at the 180th and 240th seconds were chosen to correct the baseline. The area below the baseline and the signal was calculated separately. The corrected peak area was calculated by subtracting the area below the baseline from the peak area, with the command below:

baselinearea = 0.5*(data[[180, 2]] + data[[240, 2]])*60;
*(*Find the area of baseline*)*
peakarea = Total[data[[180 ;; 240, 2]]];
*(*Find the uncorrected peak area by suming the voltage between 180s to 240s*)*
correctedarea = peakarea - baselinearea
*(*Find the corrected peak area*)*
